# Changes in the Frequency of Actions Associated With Mental Health During Online Treatment: Analysis of Demographic and Clinical Factors

**DOI:** 10.2196/57938

**Published:** 2024-07-25

**Authors:** Madelyne Bisby, Lauren Staples, Blake Dear, Nickolai Titov

**Affiliations:** 1 MindSpot Clinic MQ Health Macquarie University Sydney Australia; 2 School of Psychological Sciences Macquarie University Sydney Australia

**Keywords:** anxiety, depression, daily actions, treatments, personalization, mental health, digital treatment, analysis, clinical factors, questionnaire, depression symptoms, anxiety symptoms, patients, Australian, Australia, digital psychology service, psychology, symptom severity, severity

## Abstract

**Background:**

Specific daily actions (eg, goal setting, meaningful activities) are associated with mental health. Performing specific daily actions at a higher frequency is associated with significantly lower baseline symptoms of depression and anxiety, as well as better psychological treatment outcomes for depression and anxiety.

**Objective:**

This study explored how the frequency of specific daily actions associated with mental health may differ prior to, during, and following treatment according to demographic and clinical characteristics.

**Methods:**

Using a sample of 448 patients from an Australian national digital psychology service, we examined baseline differences in daily action frequency and changes in daily action frequency during a digital psychological treatment according to demographic and clinical subgroups. A total of 5 specific types of daily actions were measured using the Things You Do Questionnaire: healthy thinking, meaningful activities, goals and plans, healthy habits, and social connections.

**Results:**

The frequency of daily actions differed according to employment status (largest *P*=.005) and educational level (largest *P*=.004). Daily action frequency was lower in those participants with more severe or chronic depression or anxiety symptoms (largest *P*=.004). Participants reported larger increases in how often they did these daily actions from baseline to midtreatment compared to mid- to posttreatment. Depression duration (*P*=.01) and severity (*P*<.001) were associated with differences in how daily action frequency changed during treatment.

**Conclusions:**

The findings of this study support continued research exploring the relationship between daily actions and mental health, how this relationship might differ between individuals, and the clinical potential of supporting individuals to increase the frequency of daily actions to improve mental health.

## Introduction

Converging work suggests that there are different “elements,” “behaviours,” or “actions” that can be performed daily which are associated with mental health (henceforth referred to as “actions” for simplicity). Several research groups have attempted to identify these actions, such as the 5 types of daily actions on the “Things You Do” (TYD)—for example, healthy thinking and meaningful activities [1]; the “Act Belong Commit” campaign—for example, developing a sense of belonging [2]; the “ABCs of Mental Health”—for example, doing something actively [3]; and the PERMA model of well-being—for example, positive emotions and engagement [4]. While these attempts have taken different approaches to identifying these actions (eg, clinical judgment and survey methodology), they are all based on the premise that doing certain daily actions more frequently is associated with better mental health.

This premise is supported by research in both community and clinical samples. In community samples, performing some specific daily actions (eg, social and recreational activities) more frequently may prevent the onset of mental health difficulties [5]. Furthermore, the 5 types of daily actions captured by the Things You Do Questionnaire (TYDQ; healthy thinking, meaningful activities, goals and plans, healthy habits, and social connections) are associated with lower depression, lower anxiety, and greater life satisfaction in large community surveys [1].

These specific daily actions are arguably foundational to several psychological treatment approaches. For example, cognitive therapy relies on people increasing how often they respond to negative thoughts with thought challenging [6], and behavioral activation relies on people increasing how often they plan and participate in enjoyable and meaningful activities [7]. Studies that track the relationship between how often individuals practice these daily skills with treatment outcomes demonstrate that doing them more often is associated with lower distress [8,9]. Similar results are found when the frequency of specific daily actions that have been previously associated with mental health, as measured using the TYDQ, are tracked across transdiagnostic psychological treatment for depression and anxiety. Indeed, individuals report increasing how often they do these daily actions from baseline to posttreatment (8 weeks later), and those who reported doing the actions at least 3 to 4 times per week at posttreatment reported significantly lower depression and anxiety symptoms [10-12]. These findings suggest that specific daily actions are associated with mental health across the severity spectrum and that changes in how often individuals are performing these actions may influence psychological treatment outcomes.

There are a number of outstanding questions regarding individual differences in the frequency of daily actions associated with mental health. Identifying the individual characteristics associated with the specific type of daily action, and how often it is performed, could facilitate a more nuanced understanding of the relationship between these different actions and mental health. Such information could be used to identify who may benefit from interventions designed to increase the frequency of specific actions. In addition, previous work examining the rate at which psychological symptoms change over treatment has demonstrated that individuals report more rapid improvements in the first few weeks of treatment [13,14]. Preliminary research suggests that a similar trajectory may be observed for treatment-related increases in daily actions, such that individuals report more rapid increases in the frequency of daily actions within the first few weeks of treatment [11]. However, this finding needs to be replicated. To answer these questions, we analyzed an observational cohort of 448 adult patients who accessed an 8-week digital psychological treatment for depression and anxiety at an Australian digital psychology service. Specifically, we examined how the frequency of 5 types of daily actions, as measured using the TYDQ, differed between demographic and clinical subgroups prior to treatment, and then how the frequency of these daily actions changed over time according to demographic and clinical subgroups.

## Methods

### Participants

This uncontrolled cohort study included Australian adults who engaged with the MindSpot Clinic between November 2021 and May 2022. The MindSpot Clinic is a digital psychology service funded by the Australian Department of Health to provide remotely delivered psychological assessment and treatments to Australian adults [15]. Australian residents aged 18 years or older were eligible for MindSpot services. To be eligible for inclusion in this study, patients had to (1) complete the TYDQ during the initial assessment (note that this was optional) and (2) complete at least 1 lesson of a digital 8-week treatment for depression and anxiety. This resulted in a sample of 448 patients.

### Ethical Considerations

The use of deidentified data obtained from the MindSpot Clinic is approved by the Macquarie University Human Research Ethics Committee and is registered with the Australian and New Zealand Clinical Trials Registry (ACTRN12613000407796).

### Treatment

The Wellbeing Course is an 8-week digital transdiagnostic treatment course designed to help manage symptoms of depression and anxiety. The Wellbeing Course has been demonstrated to be effective for a wide range of clinical presentations [15-18]. The Wellbeing Course includes 5 lessons, practical guides, illustrative case stories, and additional resources. Lessons are released sequentially and include information and skills related to psychoeducation, cognitive challenges, physical symptom management, graded exposure, and relapse prevention. The Wellbeing Course was delivered primarily in a therapist-guided capacity, whereby therapists initiated contact with patients once a week via telephone or secure messaging. The Wellbeing Course and the clinical model provided at the MindSpot Clinic are described in detail elsewhere [15,18].

### Measures

#### Things You Do Questionnaire-15 Item

The Things You Do Questionnaire-15 (TYDQ-15) is a brief 15-item measure of daily actions that are associated with mental health [19]. There are 5 domains of action—healthy thinking, meaningful activities, goals and plans, healthy habits, and social connections. Items are scored from 0 (not at all in the last week) to 4 (every day in the last week). TYD total scores equal to performing the actions at least 3 to 4 times a week have been associated with significantly lower depression and anxiety symptoms [10,11]. The measure has demonstrated acceptable psychometric properties and treatment responsivity [19].

#### Patient Health Questionnaire-9 Item

The Patient Health Questionnaire-9 (PHQ-9) is a 9-item measure of depressive symptoms consistent with diagnostic criteria for major depression [20]. Items are scored from 0 (not at all) to 3 (all of the time); a total score of ≥10 is most frequently used as an indicator of clinical depression.

#### Generalized Anxiety Disorder-7 Item

The Generalized Anxiety Disorder-7 (GAD-7) is a 7-item measure of anxiety symptoms [21]. Items are scored from 0 (not at all) to 3 (all of the time); a total score of ≥10 is indicative of clinical anxiety.

### Statistical Analyses

Univariate analyses of variance were conducted to examine differences in action frequency according to demographic and clinical characteristics. Subgroups with fewer than 10 patients are reported in the overall demographics but were not included in analyses. For treatment analyses, 4 time points were included—initial assessment, pretreatment (week 1), midtreatment (weeks 4 and 5), and posttreatment (weeks 8 and 9). Multiple imputation was used to handle missing data consistent with intent-to-treat. The model involved a maximum of 50 case draws, 2 parameter draws, and baseline symptom severity, time, and adherence were included as predictors of missingness [22,23].

Generalized estimating equations with a gamma log-link function and unstructured correlation matrix were used to examine changes in TYD action frequency, depression symptoms, and anxiety symptoms over time [24]. Pairwise comparisons were used to examine change between consecutive time points. Demographic and clinical subgroups were then entered as predictors in the generalized estimating equations to examine changes in TYDQ scores over time according to baseline characteristics. To adjust for multiple comparisons, α was reduced to *P*<.005 for all analyses. We adopted this *P* value to balance the risk of type I and type II errors, and we acknowledge that these analyses are exploratory in nature.

## Results

### Baseline Demographic and Clinical Characteristics

Patient ages ranged from 22 to 67 years, with 255 (57%) patients between the ages of 30 and 45 years. Most participants (n=319, 71%) identified as female, lived in a capital city or surrounding area (n=275, 61%), were employed (n=352, 79%), and had received a university education (n=242, 54%). Half (n=235, 52%) of the patients were in a domestic partnership—that is, married or de facto; 339 (76%) were born in Australia; and 220 (49%) had previously received mental health treatment. The patient sample reported clinically significant symptoms: 323 (72%) reported at least moderate depressive symptoms and 294 (65%) reported at least moderate anxiety symptoms. Patients also reported relatively chronic mental health difficulties: 171 (38%) reported experiencing depressive symptoms for over 12 months and 260 (58%) reported experiencing anxiety symptoms for over 12 months.

### Baseline Characteristics and TYD Action Frequency

The frequency of daily actions on the TYDQ-15 did not differ according to age, sex, location, marital status, whether participants were born in Australia, the duration of anxiety symptoms, or mental health treatment usage (*P*s>.005; see Multimedia Appendix 1). Employment status was associated with significant differences in the frequency of total TYD actions (*P*=.002), healthy habits (*P*=.004), and social connections (*P*=.001). Participants who reported being employed reported doing daily actions at the highest frequency. Educational level was associated with differences in the frequency of total daily actions (*P*<.001), meaningful activities (*P*<.001), and goals and plans (*P*=.005). Participants who had received a tertiary education reported doing daily actions at the highest frequency. Depressive severity and duration were associated with significant differences in the total TYDQ-15 score and all domains (largest *P*=.003). Across all domains, more severe or chronic depressive symptoms were associated with performing daily actions less frequently. Anxiety severity was associated with differences in the frequency of total daily actions (*P*=.004), healthy thinking (*P*<.001), and meaningful activities (*P*=.004). Similarly, more severe or chronic anxiety symptoms were associated with performing daily actions less frequently.

### Change in Outcomes Over Time

The frequency of daily actions increased from initial assessment to posttreatment (*P*<.001). By midtreatment, 89% of the total prepost change had been realized (*d*=0.61), with a small increase observed from mid- to posttreatment (*d*=0.69; see Tables 1 and 2).

The largest increase in frequency was observed for healthy thinking, meaningful activities, and goals and plans factors. Specifically, between 85% and 97% of the prepost change in these factors had been realized by midtreatment. Within-group effect sizes increased from midtreatment (*d*s=0.56-0.61) to posttreatment (*d*s=0.58-0.62). Smaller overall increases were observed for the healthy habits and social connections factors. By midtreatment, between 81% and 93% of the total change had been reported (*d*s=0.28-0.33) with small to medium effect sizes observed at posttreatment (*d*s=0.29-0.44).

Patients reported significant reductions in depressive and anxiety symptoms from initial assessment to posttreatment (*P*s<.001). Patients had reported substantial reductions in symptoms by midtreatment (*d*s=0.72-0.76), representing 84%-89% of their total symptom reductions achieved by posttreatment. Further reductions in depressive and anxiety symptoms were observed at posttreatment (*d*s=0.72-0.74).

**Table 1:** Estimated marginal mean (SE) for treatment outcomes.

	Assessment, mean (SE)	Pretreatment, mean (SE)	Midtreatment, mean (SE)	Posttreatment, mean (SE)
PHQ-9^a^	13.56 (0.29)	12.05 (0.28)	8.90 (0.32)	8.32 (0.39)
GAD-7^b^	12.27 (0.25)	11.15 (0.25)	8.26 (0.25)	7.48 (0.35)
TYDQ-15^c^	20.72 (0.46)	23.94 (0.50)	26.59 (0.45)	27.32 (0.45)
Thoughts	4.40 (0.12)	5.12 (0.12)	5.77 (0.11)	5.94 (0.13)
Activity	3.24 (0.11)	3.97 (0.12)	4.71 (0.13)	4.76 (0.12)
Goals	3.46 (0.12)	4.23 (0.13)	4.80 (0.13)	5.04 (0.12)
Routine	4.99 (0.13)	5.45 (0.13)	5.89 (0.13)	6.10 (0.11)
Social	4.62 (0.14)	5.18 (0.14)	5.43 (0.13)	5.49 (0.14)

^a^PHQ-9: Patient Health Questionnaire-9.

^b^GAD-7: Generalized Anxiety Disorder-7.

^c^TYDQ-15: Things You Do Questionnaire-15.

**Table 2:** Metrics of clinical change in treatment outcomes.

	*P_Ass to Mid_*	Cohen *d* (95% CI)	Percentage change (95% CI)	*P_Ass to Post_*	Cohen *d* (95% CI)	Percentage change (95% CI)
PHQ-9^a^	<.001	0.72 (0.59-0.86)	34 (28-41)	<.001	0.72 (0.58-0.85)	39 (32-46)
GAD-7^b^	<.001	0.76 (0.62-0.89)	33 (27-38)	<.001	0.74 (0.61-0.88)	39 (32-46)
TYDQ-15^c^	<.001	0.61 (0.47-0.74)	28 (22-34)	<.001	0.69 (0.55-0.82)	32 (26-28)
Thoughts	<.001	0.56 (0.43-0.70)	31 (24-38)	<.001	0.58 (0.45-0.71)	35 (27-43)
Activity	<.001	0.58 (0.44-0.71)	45 (35-56)	<.001	0.62 (0.49-0.76)	47 (37-57)
Goals	<.001	0.51 (0.37-0.64)	39 (29-49)	<.001	0.62 (0.49-0.76)	46 (36-55)
Routine	<.001	0.33 (0.19-0.46)	18 (11-25)	<.001	0.44 (0.30-0.57)	22 (16-29)
Social	<.001	0.28 (0.15-0.41)	18 (9-26)	<.001	0.29 (0.16-0.42)	19 (10-27)

^a^PHQ-9: Patient Health Questionnaire-9.

^b^GAD-7: Generalized Anxiety Disorder-7.

^c^TYDQ-15: Things You Do Questionnaire-15.

### Change in TYD Action Frequency According to Demographic and Clinical Subgroups

The group x time interactions examining change in daily actions were nonsignificant for location, sex, employment status, education level, marital status, anxiety symptom severity, or the duration of their anxiety symptoms (*P*s>.025). Change in daily actions over time differed according to depression symptom severity (*P*<.001), such that those participants with more severe depressive symptoms showed larger increases in daily action frequency (see Multimedia Appendix 2 for estimated marginal means). Change in daily actions over time also differed significantly according to the duration of depression symptoms (*P*=.01). The relationship between depression duration and increases in daily action frequency was not obviously linear; rather, the largest increases were reported by those experiencing depressive symptoms for 2 weeks or less, and the smallest increases were reported by those who reported no depressive symptoms.

## Discussion

This study examined the association between individual characteristics and the frequency of daily actions prior to, during, and following a digital psychological treatment for depression and anxiety. The frequency of daily actions has been linked with mental health status—performing the specific actions measured by the TYDQ-15 at least 3 to 4 times per week is associated with significantly lower depression and anxiety symptoms [1,10,11]. Our findings replicate and extend previous work by illustrating that the majority of the change in daily action frequency (81%-97%) happens within the first month of treatment and that there may be particular groups of people who are likely to be performing daily actions less frequently or to experience the largest increases in daily action frequency after treatment. Our findings provide further evidence supporting the clinical use of assessing the frequency of daily actions as an indicator of mental health status.

A number of demographic and clinical characteristics were examined in this study, including age, sex, location, employment status, educational level, marital status, whether participants were born in Australia, whether participants reported identifying as Indigenous, the duration and severity of depressive symptoms, the duration and severity of anxiety symptoms, and the use of mental health treatment services. Being unemployed, not having a tertiary qualification, more severe or chronic depression symptoms, more severe or chronic anxiety symptoms, or experiencing depression symptoms for a longer period of time were associated with a lower frequency of actions at assessment. However, it is important to acknowledge that these analyses are exploratory in nature, and further confirmation is required before drawing conclusions regarding the impact of these individual characteristics. Nevertheless, the results do suggest that those with more chronic or severe mental health symptoms are at risk of performing daily actions less frequently.

We also examined how changes in daily actions during an 8-week digital treatment for depression and anxiety might differ according to these baseline demographic and clinical characteristics. Unlike at assessment, in which we observed that several baseline characteristics were associated with differences in baseline daily action frequency, change in daily actions over time was only impacted by the severity and duration of depressive symptoms. Indeed, the largest increases in daily action frequency were reported by those with the most severe depressive symptoms. It is not surprising that the individuals who report the largest improvements in depressive symptoms also reported the largest increases in daily action frequency; indeed, the TYDQ-15 includes items that capture actions that are negatively impacted in depression and improve with treatment such as doing enjoyable activities and having daily routines. In contrast, the relationship between the chronicity of depression symptoms and change in daily action frequency was not clear. The statistical analysis is likely to have been impacted by the small sample size and large variance in 1 subgroup (2 weeks or less; n=11). Further research is needed to understand how the duration of depression symptoms impacts changes in daily action frequency after psychological treatment.

The daily actions explored in this study overlap with those considered to be positive or adaptive coping behaviors. Positive coping refers to an individual’s ability to respond to stressors and includes behaviors such as seeking support from social networks, planning, cognitive reframing, and being physically active [25,26]. Of particular relevance to this study, research has found that positive coping is negatively associated with psychological distress [27-29]. For instance, positive coping (measured by self-efficacy and positive reappraisal) was negatively associated with subsequent psychological distress 6 months later within a COVID-19 context [30]. Future research may consider further exploring the overlap between positive coping behaviors (eg, measured by the Brief-COPE inventory [31]) and daily actions (eg, measured by the TYDQ-15), as well as how daily actions and positive coping behaviors interact to impact mental health for different individuals.

This study is not without limitations. First, as is inherent in observational cohort studies, we did not have a control group and cannot delineate the contribution of the treatment and nontreatment factors to the observed changes in daily action frequency. It is particularly important to have control groups in future research in this area, particularly when examining whether changes in daily actions are causally related to changes in psychological symptoms during treatment. Second, a cut-off score of 10 or greater was used as an indicator of clinical depression or anxiety symptoms. Although a score of 10 was recommended by the scale developers and is the most commonly used cutoffs [20,21,32,33], more contemporary research has criticized the use of such an approach [34]. There is evidence to support using a cut-off score of as low as 7 on the GAD-7 [33] and up to 14 on the PHQ-9 in general populations [35]. Considering these differences in how self-report measures capture clinical severity, future research in research settings with greater resource capacity may replicate our findings using diagnostic interviews.

This study illustrated that individuals experience rapid changes in how often they perform daily actions during psychological treatment, with up to 97% of the total change occurring within the first 4 weeks, although it is unclear whether the observed increases would be maintained without the ongoing support that was provided during treatment. These changes were prompted by the provision of information and skills founded in cognitive behavior principles, including psychoeducation around the nature of depression and anxiety (lesson 1, week 1), as well as thinking styles and cognitive challenges (lesson 2, week 2). Of course, due to the lack of a control group, it is unknown how much of the change in daily actions were a result of nontreatment factors (eg, contact with a credible health service and the passage of time). Nevertheless, these findings suggest that interventions and public campaigns that are carefully designed to support people to increase how often they perform healthy daily actions may result in improved mental health. First, the Skills for Psychological Recovery program for trauma-related mental health difficulties is based on 6 core skills (eg, helpful thinking, healthy social connections, and positive activities) and preliminary evidence suggests that it is associated with reduced psychological distress [36]. Second, the Act Belong Commit is a lifestyle framework based on 3 types of activities—keeping active, developing a sense of belonging, and doing meaningful activities [2]. The public health campaign has been associated with increased help-seeking behavior [37], and the impact of the campaign on mental health symptoms has yet to be evaluated. Third, we recently developed a 1-lesson unguided digital intervention based on daily actions. In a randomized controlled trial, the intervention was associated with reduced depression and anxiety for up to 3 months later [12]. Action-based interventions may offer a way of providing rapid and scalable mental health support outside the context of traditional psychological treatments.

Our current understanding of the individual differences in how modifiable, daily actions impact changes in mental health is underdeveloped. This study explored how demographic and clinical characteristics are associated with the frequency of daily actions prior to, during, and following a digital psychological treatment for depression and anxiety. We found that a number of baseline characteristics were associated with how often people were performing daily actions prior to beginning psychological treatment; however, only depression severity and duration were associated with differences in how these actions changed over time. Our findings provide further evidence supporting the relationship between daily actions and mental health, as well as clear directions for future research that builds on these findings.

## Appendix

Multimedia Appendix 1 Mean (SE) of Things You Do Questionnaire–15 total and factor scores at assessment according to demographic and clinical subgroups.

Multimedia Appendix 2 Estimated marginal means (SE) of overall daily actions according to depression severity and duration.

## Abbreviations

GAD-7: Generalized Anxiety Disorder-7
PHQ-9: Patient Health Questionnaire-9
TYD: Things You Do
TYDQ: Things You Do Questionnaire
TYDQ-15: Things You Do Questionnaire-15
