# Supplementary material for: Changes in the Frequency of Actions Associated With Mental Health During Online Treatment: Analysis of Demographic and Clinical Factors
Source: JMIR Form Res. 2024 Jul 25;8:e57938. doi: 10.2196/57938 (PMC11310636; doi:10.2196/57938)
Supplement: Multimedia Appendix 1 [file formative_v8i1e57938_app1.docx]

| **Sample** | **N** | **Total** | **Thinking** | | **Activity** | **Goals** | **Habits** | **Social** | |
| --- | --- | --- | --- | --- | --- | --- | --- | --- | --- |
| **Total** | 448 | 20.71 (0.46) | 4.40 (0.12) | 3.24 (0.11) | | 3.46 (0.12) | 4.99 (0.13) | 4.63 (0.14) | |
|  |  |  |  |  | |  |  |  | |
| **Age** |  | ns | ns | ns | | ns | ns | ns | |
| 18 – 29 years | 79 | 19.51 (1.10) | 3.80 (0.29) | 3.14 (0.27) | | 3.25 (0.30) | 4.41 (0.31) | 4.91 (0.33) | |
| 30 – 45 years | 255 | 21.38 (0.61) | 4.63 (0.16) | 3.33 (0.15) | | 3.60 (0.17) | 5.12 (0.17) | 4.69 (0.19) | |
| 46 – 60 years | 107 | 20.13 (0.95) | 4.34 (0.25) | 3.10 (0.23) | | 3.31 (0.26) | 5.11 (0.26) | 4.27 (0.29) | |
| 61+ years ^a^ | 7 | 19.14 (3.70) | 3.43 (0.98) | 3.14 (0.89) | | 2.71 (1.00) | 5.29 (1.03) | 4.57 (1.12) | |
|  |  |  |  |  | |  |  |  | |
| **Gender** |  | ns | ns | ns | | ns | ns | ns | |
| Male | 126 | 20.52 (0.87) | 4.28 (0.23) | 3.23 (0.21) | | 3.46 (0.23) | 5.10 (0.25) | 4.45 (0.27) | |
| Female | 319 | 20.74 (0.55) | 4.44 (0.14) | 3.23 (0.13) | | 3.44 (0.15) | 4.95 (0.15) | 4.68 (0.16) | |
| Other ^a^ | 3 | 26.00 (5.65) | 5.00 (1.51) | 4.67 (1.36) | | 5.33 (1.52) | 5.00 (1.58) | 6.00 (1.71) | |
|  |  |  |  |  | |  |  |  | |
| **Location** |  | ns | ns | ns | | ns | ns | ns | |
| Capital city or surrounds | 275 | 21.45 (0.59) | 4.51 (0.16) | 3.40 (0.14) | | 3.65 (0.16) | 5.10 (0.17) | 4.79 (0.18) | |
| Other urban region | 79 | 17.95 (1.09) | 4.15 (0.29) | 2.65 (0.26) | | 2.75 (0.29) | 4.44 (0.31) | 3.96 (0.33) | |
| Rural or remote region | 94 | 20.73 (1.00) | 4.26 (0.27) | 3.22 (0.24) | | 3.45 (0.27) | 5.14 (0.28) | 4.67 (0.31) | |
|  |  |  |  |  | |  |  |  | |
| **Employment** |  | **.002** | ns | ns | | ns | **.004** | **.001** | |
| Employed | 352 | 21.65 (0.51) | 4.54 (0.14) | 3.40 (0.13) | | 3.58 (0.14) | 5.20 (0.14) | 4.92 (0.16) | |
| Unemployed | 40 | 15.78 (1.53) | 3.40 (0.41) | 2.40 (0.37) | | 3.00 (0.42) | 3.58 (0.43) | 3.40 (0.46) | |
| Student | 21 | 19.29 (2.11) | 4.57 (0.56) | 3.10 (0.51) | | 3.86 (0.58) | 4.76 (0.59) | 3.00 (0.64) | |
| Home duties | 25 | 18.52 (1.93) | 4.04 (0.51) | 2.80 (0.47) | | 2.64 (0.53) | 4.80 (0.54) | 4.24 (0.59) | |
| Retired ^a^ | 5 | 20.20 (4.30) | 5.20 (1.16) | 3.00 (1.04) | | 2.40 (1.18) | 6.00 (1.21) | 3.60 (1.31) | |
| Disability support/ benefit ^a^ | 5 | 12.20 (4.30) | 2.40 (1.16) | 1.60 (1.04) | | 2.20 (1.18) | 3.00 (1.21) | 3.00 (1.31) | |
|  |  |  |  |  | |  |  |  | |
| **Education** |  | **<.001** | ns | **<.001** | | **.005** | ns | ns | |
| High school or less | 69 | 18.29 (1.16) | 3.87 (0.31) | 2.75 (0.28) | | 3.04 (0.31) | 4.51 (0.33) | 4.12 (0.36) | |
| Trade certificate or diploma | 137 | 19.42 (0.82) | 4.10 (0.22) | 2.80 (0.20) | | 3.17 (0.22) | 4.75 (0.23) | 4.60 (0.25) | |
| Undergraduate degree | 124 | 20.77 (0.86) | 4.61 (0.23) | 3.37 (0.21) | | 3.32 (0.23) | 4.91 (0.24) | 4.56 (0.27) | |
| Postgraduate degree | 118 | 23.59 (0.89) | 4.82 (0.24) | 3.90 (0.21) | | 4.19 (0.24) | 5.64 (0.25) | 5.03 (0.27) | |
|  |  |  |  |  | |  |  |  | |
| **Marital status** |  | ns | ns | ns | | ns | ns | ns | |
| Never married | 154 | 19.66 (0.78) | 4.04 (0.21) | 3.22 (0.19) | | 3.41 (0.21) | 4.63 (0.22) | 4.36 (0.24) | |
| Domestic partnership | 235 | 21.97 (0.63) | 4.61 (0.17) | 3.44 (0.15) | | 3.60 (0.17) | 5.36 (0.18) | 4.96 (0.19) | |
| Divorced or separated | 57 | 18.74 (1.29) | 4.53 (0.34) | 2.54 (0.31) | | 3.09 (0.35) | 4.54 (0.36) | 4.04 (0.39) | |
| Widowed ^a^ | 2 | 11.50 (5.50) | 3.00 (1.00) | 1.50 (1.50) | | 1.00 (1.00) | 2.50 (1.50) | 3.50 (0.50) | |
|  |  |  |  |  | |  |  |  | |
| **Born in Australia** | | ns | ns | ns | | ns | ns | ns | |
| No | 109 | 20.49 (0.94) | 4.61 (0.25) | 3.20 (0.23) | | 3.35 (0.25) | 5.11 (0.26) | 4.22 (0.28) | |
| Yes | 339 | 20.79 (0.53) | 4.33 (0.14) | 3.25 (0.13) | | 3.49 (0.14) | 4.96 (0.15) | 4.76 (0.16) | |
|  |  |  |  |  | |  |  |  | |
| **Depression Severity** | | **<.001** | **<.001** | **<.001** | | **<.001** | **<.001** | **<.001** | |
| Minimal | 29 | 31.10 (1.60) | 6.62 (0.45) | 5.38 (0.39) | | 5.62 (0.46) | 7.14 (0.47) | 6.35 (0.54) | |
| Mild | 96 | 26.03 (0.88) | 5.51 (0.25) | 4.52 (0.22) | | 4.51 (0.25) | 6.27 (0.26) | 5.22 (0.29) | |
| Moderate | 127 | 21.18 (0.76) | 4.47 (0.21) | 3.31 (0.19) | | 3.40 (0.22) | 5.05 (0.22) | 4.96 (0.26) | |
| Moderately Severe | 116 | 17.27 (0.80) | 3.69 (0.22) | 2.42 (0.20) | | 2.77 (0.23) | 4.46 (0.23) | 3.93 (0.27) | |
| Severe | 80 | 14.83 (0.96) | 3.15 (0.27) | 2.01 (0.24) | | 2.51 (0.28) | 3.38 (0.28) | 3.78 (0.32) | |
|  |  |  |  |  | |  |  |  | |
| **Depression Duration** | | **<.001** | **<.001** | **<.001** | | **.003** | **.001** | **<.001** | |
| Not at all | 158 | 24.75 (0.74) | 5.29 (0.20) | 4.19 (0.18) | | 4.08 (0.21) | 5.66 (0.21) | 5.53 (0.23) | |
| 2 weeks or less | 11 | 21.18 (2.81) | 5.36 (0.76) | 2.46 (0.68) | | 3.82 (0.78) | 5.00 (0.81) | 4.55 (0.87) | |
| 2 weeks to 6 months | 70 | 18.44 (1.11) | 3.90 (0.30) | 2.80 (0.27) | | 2.67 (0.31) | 4.59 (0.32) | 4.49 (0.35) | |
| 6-12 months | 38 | 20.61 (1.51) | 4.18 (0.41) | 3.05 (0.37) | | 3.32 (0.42) | 5.37 (0.44) | 4.68 (0.47) | |
| 1-5 years | 78 | 17.72 (1.06) | 3.85 (0.29) | 2.44 (0.26) | | 2.97 (0.29) | 4.73 (0.31) | 3.73 (0.33) | |
| 5-10 years | 26 | 20.23 (1.83) | 3.96 (0.49) | 2.89 (0.44) | | 3.89 (0.51) | 4.85 (0.53) | 4.65 (0.57) | |
| 10+ years | 67 | 17.22 (1.14) | 3.57 (0.31) | 2.78 (0.28) | | 3.22 (0.32) | 4.00 (0.33) | 3.66 (0.35) | |
|  |  |  |  |  | |  |  |  | |
| **Anxiety Severity** |  | **.004** | **<.001** | **.004** | | ns | ns | ns | |
| Minimal | 26 | 23.27 (1.90) | 5.62 (0.50) | 3.65 (0.45) | | 4.27 (0.51) | 5.54 (0.53) | 4.19 (0.58) | |
| Mild | 128 | 22.72 (0.85) | 5.15 (0.22) | 3.80 (0.21) | | 3.84 (0.23) | 5.21 (0.24) | 4.72 (0.26) | |
| Moderate | 133 | 20.60 (0.84) | 4.20 (0.22) | 3.14 (0.20) | | 3.42 (0.23) | 5.26 (0.24) | 4.59 (0.26) | |
| Severe | 161 | 18.80 (0.76) | 3.76 (0.20) | 2.82 (0.18) | | 3.05 (0.21) | 4.51 (0.22) | 4.66 (0.23) | |
|  |  |  |  |  | |  |  |  | |
| **Anxiety Duration** | | ns | ns | ns | | ns | ns | ns | |
| Not at all | 42 | 22.02 (1.51) | 5.14 (0.40) | 3.36 (0.36) | | 3.74 (0.41) | 5.64 (0.42) | 4.14 (0.46) | |
| 2 weeks or less | 10 | 19.20 (3.10) | 3.70 (0.82) | 2.90 (0.75) | | 2.40 (0.83) | 4.10 (0.87) | 6.10 (0.94) | |
| 2 weeks to 6 months | 83 | 21.75 (1.08) | 4.57 (0.29) | 3.37 (0.26) | | 3.59 (0.30) | 5.06 (0.30) | 5.16 (0.33) | |
| 6-12 months | 53 | 21.21 (1.35) | 4.66 (0.36) | 3.02 (0.32) | | 3.57 (0.36) | 5.32 (0.38) | 4.64 (0.41) | |
| 1-5 years | 111 | 19.81 (0.93) | 4.30 (0.25) | 3.09 (0.22) | | 3.10 (0.25) | 4.87 (0.26) | 4.45 (0.28) | |
| 5-10 years | 44 | 21.82 (1.48) | 4.27 (0.39) | 3.61 (0.36) | | 4.02 (0.40) | 5.00 (0.41) | 4.91 (0.45) | |
| 10+ years | 105 | 19.76 (0.96) | 4.05 (0.25) | 3.24 (0.23) | | 3.43 (0.26) | 4.72 (0.27) | 4.32 (0.29) | |
|  |  |  |  |  | |  |  |  | |
| **Mental Health Treatment** | | ns | ns | ns | | ns | ns | ns | |
| Never | 110 | 21.34 (0.93) | 4.76 (0.25) | 3.44 (0.22) | | 3.34 (0.25) | 5.26 (0.26) | 4.55 (0.28) | |
| Previous | 220 | 20.78 (0.66) | 4.44 (0.18) | 3.22 (0.16) | | 3.56 (0.18) | 5.11 (0.18) | 4.44 (0.20) | |
| Current | 118 | 20.02 (0.90) | 3.98 (0.24) | 3.09 (0.22) | | 3.38 (0.24) | 4.53 (0.25) | 5.05 (0.27) | |
| ***Note.*** ns = non-significant. ^a^ Not included in statistical comparison due to small sample size. | | | | | | | | |  |
