# Supplementary material for: Changes in the Frequency of Actions Associated With Mental Health During Online Treatment: Analysis of Demographic and Clinical Factors
Source: JMIR Form Res. 2024 Jul 25;8:e57938. doi: 10.2196/57938 (PMC11310636; doi:10.2196/57938)
Supplement: Multimedia Appendix 2 [file formative_v8i1e57938_app2.docx]

|  |  | **Mean (SE)** | | | | ***Ass to Mid*** | | ***Ass to Post*** | | | |
| --- | --- | --- | --- | --- | --- | --- | --- | --- | --- | --- | --- |
|  | **N** | **Ass** | **Pre** | **Mid** | **Post** | **Cohen’s d** | **Percentage change** | **Cohen’s d** | | **Percentage change** | |
| **Depression Severity** | | |  |  |  |  |  |  | |  | |
| Minimal | 29 | 31.10 (1.45) | 32.31 (1.67) | 32.42 (1.54) | 33.68 (1.68) | 0.16  (-0.35, 0.68) | 4  (-9, 18) | | 0.31  (-0.22, 0.82) | | 8  (-6, 23) |
| Mild | 96 | 26.03 (1.01) | 28.96 (1.07) | 29.21 (1.14) | 29.20 (1.09) | 0.30  (0.02, 0.58) | 12  (1, 24) | 0.31  (0.02, 0.59) | | 12  (1, 23) | |
| Moderate | 127 | 21.18 (0.74) | 24.35 (0.82) | 27.36 (0.81) | 28.51 (0.88) | 0.71  (0.45, 0.96) | 29  (19, 39) | 0.80  (0.54, 1.05) | | 35  (24, 45) | |
| Moderately Severe | 116 | 17.27 (0.70) | 21.00 (0.84) | 24.34 (0.75) | 25.56 (0.75) | 0.90  (0.63, 1.17) | 41  (29, 53) | 1.06  (0.78, 1.33) | | 48  (36, 60) | |
| Severe | 80 | 14.83 (0.95) | 18.55 (1.21) | 23.36 (1.02) | 23.42 (0.86) | 0.97  (0.64, 1.29) | 58  (39, 76) | 1.06  (0.72, 1.39) | | 58  (41, 75) | |
|  |  |  |  |  |  |  |  |  | |  | |
| **Depression Duration** | | |  |  |  |  |  |  | |  | |
| Not at all | 158 | 24.75 (0.81) | 27.41 (0.83) | 28.51 (0.78) | 29.38 (0.77) | 0.38  (0.15, 0.60) | 15  (6, 24) | 0.47  (0.24, 0.69) | | 19  (10, 28) | |
| 2 weeks or less | 11 | 21.18 (2.71) | 27.18 (3.25) | 33.28 (2.76) | 30.66 (2.83) | 1.33  (0.36, 2.20) | 57  (19, 95) | 1.03  (0.10, 1.88) | | 45  (6, 83) | |
| 2 weeks to 6 months | 70 | 18.44 (0.92) | 22.40 (1.17) | 26.30 (1.11) | 28.10 (1.19) | 0.92  (0.57, 1.26) | 43  (27, 58) | 1.09  (0.72, 1.43) | | 52  (36, 69) | |
| 6-12 months | 38 | 20.61 (1.56) | 23.13 (1.52) | 26.59 (1.62) | 27.04 (1.55) | 0.61  (0.14, 1.06) | 29  (7, 51) | 0.67  (0.14, 1.06) | | 31  (10, 52) | |
| 1-5 years | 78 | 17.72 (0.99) | 22.00 (1.22) | 24.75 (1.15) | 24.35 (1.10) | 0.74  (0.41, 1.06) | 40  (23, 57) | 0.72  (0.39, 1.04) | | 37  (21, 54) | |
| 5-10 years | 26 | 20.23 (1.63) | 23.77 (1.89) | 24.72 (1.61) | 26.84 (1.57) | 0.54  (-0.02, 1.09) | 22  (-1, 45) | 0.81  (23, 1.36) | | 33  (10, 55) | |
| 10+ years | 67 | 17.22 (1.12) | 19.67 (1.22) | 24.11 (1.17) | 24.92 (1.09) | 0.73  (0.38, 1.08) | 40  (21, 59) | 0.85  (0.49, 1.20) | | 45  (27, 63) | |
